# Measuring the Bright Side of Being Blue: A New Tool for Assessing Analytical Rumination in Depression

**DOI:** 10.1371/journal.pone.0112077

**Published:** 2014-11-14

**Authors:** Skye P. Barbic, Zachary Durisko, Paul W. Andrews

**Affiliations:** 1 Social Aetiology of Mental Illness (SAMI) Canadian Institute of Health Research (CIHR) Training Program, Centre for Addiction and Mental Health, Toronto, Ontario, Canada; 2 Department of Psychology, Neuroscience & Behaviour, McMaster University, Hamilton, Canada; Iranian Institute for Health Sciences Research, ACECR, Islamic Republic of Iran

## Abstract

**Background:**

Diagnosis and management of depression occurs frequently in the primary care setting. Current diagnostic and management of treatment practices across clinical populations focus on eliminating signs and symptoms of depression. However, there is debate that some interventions may pathologize normal, adaptive responses to stressors. Analytical rumination (AR) is an example of an adaptive response of depression that is characterized by enhanced cognitive function to help an individual focus on, analyze, and solve problems. To date, research on AR has been hampered by the lack of theoretically-derived and psychometrically sound instruments. This study developed and tested a clinically meaningful measure of AR.

**Methods:**

Using expert panels and an extensive literature review, we developed a conceptual framework for AR and 22 candidate items. Items were field tested to 579 young adults; 140 of whom completed the items at a second time point. We used Rasch measurement methods to construct and test the item set; and traditional psychometric analyses to compare items to existing rating scales.

**Results:**

Data were high quality (<1% missing; high reliability: Cronbach's alpha  = 0.92, test-retest intraclass correlations >0.81; evidence for divergent validity). Evidence of misfit for 2 items suggested that a 20-item scale with 4-point response categories best captured the concept of AR, fitting the Rasch model (χ^2^ = 95.26; df  = 76, *p* = 0.07), with high reliability (*r_p_* = 0.86), ordered response scale structure, and no item bias (gender, age, time).

**Conclusion:**

Our study provides evidence for a 20-item Analytical Rumination Questionnaire (ARQ) that can be used to quantify AR in adults who experience symptoms of depression. The ARQ is psychometrically robust and a clinically useful tool for the assessment and improvement of depression in the primary care setting. Future work is needed to establish the validity of this measure in people with major depression.

## Introduction

Depression affects approximately 350 million people worldwide and is a leading cause of global disability [1], [2]. Alleviating depression assumes ever increasing importance as the individual and societal costs associated with depression rise every day [3]. Depression is associated with factors that increase mortality risk such as poor adherence to medical treatment and self-care for diabetes and cardiovascular disease [4] [5], health behaviors such as smoking and lack of physical activity [6], cognitive impairment [7] and disability [8]. It is also a common consequence of changes in health status (i.e., cancer [9] & stroke [10]), and/or new life roles (i.e., caregiving [11], immigration [12], and loss of employment [13]).

Primary care is a frequent entry point into the health care system for depressed patients. Since the 1980's gaps in quality of depression care in primary care systems have been noted and continue to be highlighted today [3], [14]–[16]. Studies show that only 25% to 50% of patients with depression are accurately diagnosed by primary care physicians and, among those who are accurately diagnosed, few receive the recommended dosage and duration of either pharmacotherapy or evidence-based psychotherapy [16], [17]. Confusing the picture, the medical community receives conflicting accounts of subclinical symptoms. Some argue that subclinical and clinical episodes are part of a single pathological continuum that should often be treated with medication [18], while others argue subclinical symptoms are often a normal response to stress [19].

In short, greater understanding of both clinical and subclinical depression will help primary care physicians, who are often the first line of treatment for depression, improve the overall health and quality of life of their patients.

### Why does depression exist?

Despite decades of research, the molecular and physiological mechanisms underlying depression are not fully understood [20]–[22]. In addition, there is ongoing debate about the safety and efficacy of pharmacological and psychological treatments [23]–[28]. While efforts continue to understand *how* people become depressed, research from an evolutionary perspective (so-called “Darwinian Psychiatry” or “Evolutionary Medicine”) asks *why* depression exists. Evolutionary medicine seeks to understand the difference between healthy and disordered states and why humans are susceptible to disease [29], [30]. This perspective has informed our understanding of a broad range of psychiatric conditions and has been reviewed in detail previously [31], [32]. Evolutionary hypotheses of the aetiology of depression are numerous [33], [34], but typically suggest that depression has evolved as an adaptation to help regulate energy use and navigate adverse situations. If depression can indeed be adaptive, primary health care providers and researchers may need to consider different approaches to treatment.

### The Concept of Interest: Analytical Rumination

One leading hypothesis of the origin of depression proposes that many depressions are the result of an ancient defence mechanism designed by natural selection to promote analytical thinking in response to complex life stressors [35]. The *analytical rumination hypothesis* [35] states that the symptoms of depression result in extended bouts of persistent, distraction-resistant cognitive analysis, which can function to help individuals resolve challenges in their lives. This hypothesis recognizes that the resolution of exceptionally complex problems, such as those associated with adverse life events and major stressors, can require prolonged and in-depth bouts of analysis that lead to impairment and disengagement from everyday life. Problems can occur in a variety of contexts, but analysis will involve thinking through the components of the problem such as (1) its cause; (2) the aspects that need solving; (3) potential solutions; and (4) the costs and benefits associated with implementing various solutions.

While the ruminative thoughts associated with depression are commonly considered maladaptive [36]–[38], several authors have argued that depressive ruminations may be useful, or at least may begin as a useful means to focus and analyze problems in order to gain insight [39]–[41]. A substantial body of evidence indicates that depressed mood is associated with increased cognitive processing, improved accuracy on complex tasks, and enhanced detail-oriented judgement on tasks that require deliberate information processing [42]–[46]. Individuals with depression have also been shown to consistently outperform non-depressed controls when the experimental tasks involve cost-benefit analysis [47]–[52].

### Clinical implications for understanding analytical rumination

Understanding analytical rumination has important clinical implications for how to assess and treat depression. Rather than viewing depression as an impairment or malfunction of the brain, the evolutionary perspective hypothesizes that it may sometimes occur as an adaptive response to promote the cognitive analysis required to understand and resolve current problems. Depressive episodes associated with high levels of analytical rumination may be most usefully treated by facilitating rumination and analysis rather than medications or psychotherapies that may treat rumination as unproductive.

### The challenge to understanding analytical rumination

Research in this arena has been limited by the lack of a reliable and valid psychometric instrument for analytical rumination. Analytical rumination, similar to many other important health constructs (i.e, quality of life), is not directly measurable (i.e., it is *latent*). Primary health care providers must rely on patient-reported outcomes (PROs) to gain information about the patient that cannot be collected by means of traditional clinical metrics such as lab values. Recently, the use of PROs has been emphasized as a valuable means to enhance care management by helping providers to understand not just whether a clinical value is within range, but how patients' lives may be affected by the value [53]. In order to develop a PRO that can be integrated into routine care in a clinically meaningful way, development and testing needs to carefully consider the concept of interest, content of use, and measurement rigour (i.e., precision, standardization, and comparability of scores across studies and diseases) [53], [54].

Based on a thorough review of the theoretical construct [35], we are unaware of any measure that captures the full range of analytical rumination in a clinically meaningful way. The objective of this study was to develop and test a conceptually and psychometrically sound measure of analytical rumination to inform fundamental decisions in primary care practice, health research, and treatment trials.

## Methods

### Measure design

**Figure 1:**
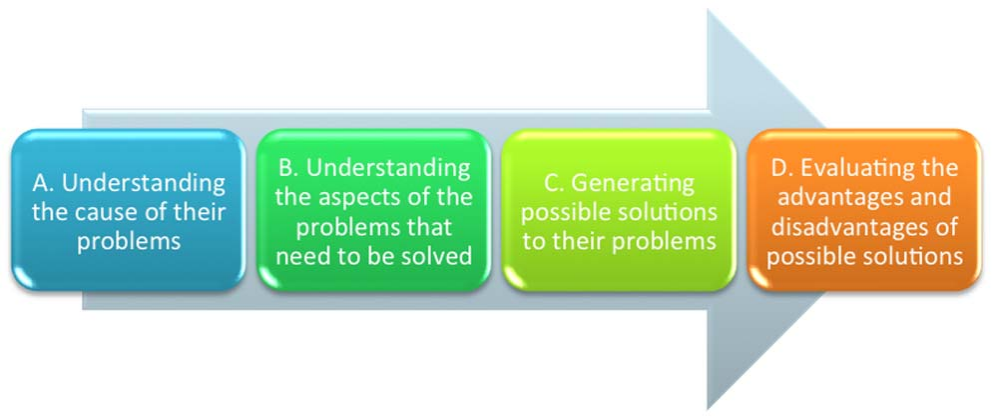
Working model describing the theoretical conceptualization of analytical rumination.

We developed a conceptual model (Figure 1) based on an extensive review of published theory on analytical rumination and depression [35]. The analytical rumination hypothesis states that individuals with depression engage in analysis to understand at least four different parts (domains) of their problems: (1) understanding the cause (e.g., “I tried to understand why I had these problems”); (2) understanding the aspects of the problems that need to be solved (e.g., “I tried to understand what was wrong in my life”; (3) generating possible solutions (e.g., “I thought about all my options for dealing with my problems”); and (4) evaluating the advantages and disadvantages of possible solutions (e.g., “I thought about whether my options for dealing with one problem would make other problems worse”). From this model, we generated 22 candidate items to capture the full range of analytical rumination, which we refer to as the Analytical Rumination Questionnaire (ARQ).

As described below, each item of the ARQ candidate item pool was scored on a 5-point Likert scale. Scoring categories range from 1 (none of the time) to 5 (all of the time). Possible scores ranged from 22–110, with a higher score indicating a higher level of analytical rumination (see Appendix S1 for candidate items in ARQ). We hypothesized that the four domains and the items themselves had a natural implicit ordering from low to high. Specifically, we hypothesized that people first attempt to understand why they have a problem (domain 1) and what needs to be solved (domain 2) before they attempt to generate (domain 3) and evaluate (domain 4) possible solutions.

### Questionnaire administration

#### Participants, recruitment, and data collection

All participants were students at McMaster University taking undergraduate psychology courses. English-speaking adults aged 18 years and over were eligible to participate. We collected data in two studies. In the first, 439 participants filled out the ARQ at one time point, and in the second 140 participants filled it out at two time points. In order to encourage high response rates, we offered academic credit for participation. Both studies were approved by the McMaster Research Ethics Board and written informed consent was obtained prior to completing the ARQ.

### Analysis Procedure

We used both traditional and Rasch psychometric analyses to evaluate the properties of the ARQ.

### Traditional analyses

Traditional psychometric analyses have been described in detail elsewhere [55]. In brief, they use correlation and descriptive analyses to evaluate scaling assumptions (legitimacy of summing items), reliability, and validity [56]. Accordingly, we examined data from the ARQ for quality (percent missing for each item), scaling assumptions, scale to sample targeting (score means; standard deviation (SD); floor and ceiling effects), and internal consistency and reliability(Cronbach's alphas) [57]. We determined convergent and discriminant construct validity by examining correlations between the ARQ and other 3 other measures and variables (age and sex). For discriminant validity testing, we used the Beck Depression Inventory (BDI) [58] and the Positive and Negative Affect Scale (PANAS) [59]. For convergent validity testing we used the reflective pondering subscale of the Ruminative Response Scale (RRS) [37], [38]. We hypothesized that correlations would be the highest with the ARQ and the reflective pondering subscale of the RRS, and the correlations of the ARQ with other variables would be lower.

### Rasch Measurement Psychometric Testing

Rasch measurement is a paradigm commonly used to guide the development and testing of rating scales. Many statistical techniques for evaluating psychometric instruments attempt to develop a model from data that describes how people use an instrument. In contrast, a fundamental goal of Rasch measurement is to develop a psychometric instrument that reflects an *a priori* specified conceptual model [60]. One component of this conceptual model is *specific objectivity* (i.e., the instrument objectively measures the latent trait in the same way that a yardstick is an instrument for objectively measuring length). A specifically objective psychometric instrument must have several properties. First, all the items of the instrument must be related to a single latent trait (i.e., the instrument must be unidimensional) [61]. Second, for each item, there must be a monotonic relationship between the ordering of the responses of that item and the ordering of the latent trait [56]. For instance, for item 1 of the ARQ, people who rank higher on the latent analytical rumination trait must be probabilistically more likely to endorse higher responses. Third, there must be local independence [62], which means that the answer to an item does not depend on the order in which items are presented. Finally, while a Rasch model allows items to differ in how diagnostic they are of the latent trait (i.e., some items indicate low levels of the latent trait while other items indicate high levels of the latent trait), the diagnostic ordering of items should not vary across the range of the latent trait [63]. For example, if a person who is low on the latent trait of analytical rumination is more likely to endorse item 1 of the ARQ than item 13, then this ordering must be preserved at higher levels of the latent trait. These assumptions are difficult to achieve in practice, so a psychometric instrument that fits the Rasch model has passed an important, rigorous test of measurement.

When a psychometric instrument satisfies the rigorous assumptions of the Rasch model, the sum of the scores of the individual items provides a complete description of the person's standing on the latent variable. An instrument that defines the full spectrum of the latent variable will range from -4 to +4 logits, corresponding to ±4 standard deviations of a standard normal distribution, and items will cover all levels of the latent distribution. Moreover, an instrument that fits the context of use is one that captures the full range of the latent distribution in a given population [64], [65]. A range of parameters arising from the Rasch analysis can be used to judge the extent to which there is misfit between the items and people on this range, and as a result, the extent to which scoring and summing items is in fact, a valid and reliable approach [66].

For this analysis, we used all 22 candidate items. All assumptions were verified using the Masters' partial credit Rasch polytomous model [67], an appropriate mathematical derivation of the Rasch model suitable for investigating items with ordinal response options. All analyses were performed using RUMM 2030 [68].

#### Clinical Meaning

We examined the extent to which ARQ items were clinically cohesive and reflected our *a priori* hypothesis about how items covered the latent spectrum of low to high analytical rumination.

#### Thresholds for item response options

Each item of the ARQ was scored on a 5-point Likert scale, with five response categories (none of the time, some of the time, half of the time, most of the time, all of the time), and five integer scores assigned to each category (1, 2, 3, 4, and 5, respectively). The successive nature of the scores implies that there is a natural order to the assignment that reflects a continuum of increasing impact from less (i.e., 1 =  not at all) to more (i.e., 5 =  all of the time). We tested this assumption by statistical and graphical inspection of threshold locations and plots.

#### Item fit statistics

We tested the extent to which the participant's responses to an item fit the rigorous expectations of the Rasch model. Misfit of an item implies that the item is not working as intended and may not be measuring the intended construct. We used three indicators of fit: (1) log residuals (item-person interaction) (2) chi-square values (item-trait interaction), and (3) item characteristic curves. Rather than using absolute criteria for interpreting fit, these three indicators of fit were interpreted separately to understand the context of their use as a full item set capturing analytical rumination.

#### Item locations and targeting

We carefully looked at how items were distributed along the proposed latent analytical rumination continuum. We flagged items in similar locations as potentially redundant and warranting further investigation. We gauged the calibration of the instrument to the population by comparing graphically how closely the amount of analytical rumination displayed by the respondents was adequately measured by the items on the scale.

#### Person Separation Index (PSI) [69]

We used the PSI as a reliability statistic, analogous to Cronbach's alpha [57], to test the extent to which scale scores in the sample can be separated. Higher scores indicate higher reliability.

#### Differential Item Functioning (DIF)

We determined whether each item's location on the latent analytical rumination construct was stable across groups using item characteristic curves and two-way analyses of variance with a Bonferroni correction of 0.05 for multiple comparisons. Groups included gender, age, ethnicity, and whether the individual reported a medical condition.

#### Unidimensionality

We tested the scale's ability to measure a single latent construct using a principal components analysis (PCA) of the residuals. We specifically tested the presence of a pattern of the residuals grouping into more than one subscale once the “Rasch factor” was extracted. We hypothesized that the response structure would be unidimensional and that, apart from a single variable and the item parameters mapped on this variable, the remaining variation was random. Depending on the factor loadings resulting from the PCA, we performed paired t-tests to assess whether person estimates derived from the subtests of items were significantly different from each other. If greater than 5% of t-tests were significant, explanation for the anomaly was put into question.

#### Dependency

We tested to see whether the responses to any of the items in the scale directly influenced the response to other items by examining item residual correlations.

## Results

The sample consisted of 308 women (53%) and 271 men (47%) at enrollment with a mean age of 19 years (SD: 1.9). Thirty percent reported being of white-European descent, followed by 16% Asian, 9% East Asian, 5% African, 2% Aboriginal, and 14% reporting “Other”. Thirty-three percent of the sample reported taking medication, with 28% of this sub-sample reporting contraceptive medication, and 7% reporting a form of anti-depressant medication.

### Traditional Psychometric Results

Data satisfied criteria for all evaluated traditional psychometric properties. Missing data from all items ranged from <1%–2%. Scale scores were computable for 99% of respondents. Scale scores spanned the range of the scale and were not notably skewed. We did not observe any ceiling and floor effects.

#### Reliability and Validity

Internal consistency reliability was high (Cronbach alphas  =  0.91), and the mean inter-item correlation was 0.83, supporting scale reliability. Scale validity was supported by the high Cronbach alpha coefficient and interscale correlations. Table 1 shows the results of the convergent and discriminant construct validity testing of the ARQ. Patterns of correlations were consistent with our predictions. Mean ARQ scores were correlated highest with the RRS subscale (r = 0.40), followed by the BDI and PANAS. As expected, the mean scores for men and women did not differ, nor did age impact ARQ scores.

**Table 1:** Traditional psychometric methods: convergent and discriminant construct validity and group differences validity.

Instrument/variable	Scale/Variable	Correlation to the ARQ
RRS- Reflective Pondering	Sub Score	0.40*
RRS- Brooding	Sub Score	0.22
BDI	Total Score	0.25
PANAS	Total Score	0.20
Demographic variables		
	Age	0.13
	Sex	0.03
	Medication	0.15

*Significant <0.05; ARQ: Analytical Rumination Questionnaire, high scores indicate greater analytical rumination; RRS: Ruminative Response Scale, high scores indicate greater rumination; BDI: Beck Depression Scale, high scores indicate greater depression; PANAS: Positive and negative affect scale.

### Rasch Measurement Results

#### Clinical Meaning

The hierarchy of the items was clinically meaningful. Most (20/22) items mapped back to the *a priori* hypothesized analytical rumination continuum, with the expected order of item difficulty capturing a theoretical distribution of low to high. Table 2 shows the ordering of the items from least to most difficult.

**Table 2:** Measures of fit and location (SE) of ARQ items.

Item	Item label	Location	SE	Fit Resid.	χ^2†^	Prob*
22	I tried to think through my difficulties	−0.642	0.059	0.509	4.991	0.288
16	I tried to learn from my mistakes	−0.550	0.057	1.719	1.354	0.852
17	I tried to find a goal or purpose that was meaningful to me	−0.511	0.057	1.539	1.112	0.892
20	I tried to find a way to resolve an important issue	−0.405	0.059	−1.498	7.203	0.126
7	I tried to figure out the best option for dealing with my dilemma	−0.309	0.060	−0.929	7.268	0.122
19	I tried to figure out how to stick to my goals	−0.292	0.058	−1.577	9.721	0.050
18	I tried to find an answer to my problems	−0.273	0.055	1.077	1.362	0.850
6	I thought about all the options for dealing with my problems	−0.269	0.062	−1.169	10.407	0.034
12	I tried to figure out how to make the best out of a bad situation	−0.137	0.054	**−2.621**	12.412	0.023
8	I tried to figure out which of the problems I was facing were the most important and which I should do first	−0.006	0.056	−0.224	1.572	0.813
21	I tried to understand the past and the present	0.036	0.073	0.811	0.575	0.966
5	I thought about all the aspects of the problems I was facing that needed to be solved	0.053	0.057	−1.116	4.979	0.297
3	I thought about what I may have done to avoid these problems	0.081	0.054	0.116	1.583	0.812
1	I tried to understand why I had these problems	0.122	0.057	−0.468	4.708	0.319
2	I tried to figure out what I had done wrong	0.230	0.055	1.440	6.227	0.183
14	I tried to figure out how to best avoid future problems	0.278	0.057	1.027	0.388	0.983
10	I thought about whether some of the options I could take were likely to solve my problems or make things worse.	0.425	0.052	2.283	9.427	0.051
4	I thought about all the ways my life had become more difficult	0.496	0.052	2.048	12.494	0.015
15	I tried to figure out what was wrong in my life	0.509	0.052	1.474	2.426	0.658
11	I thought about whether my options for dealing with one problem would make other problems worse	1.028	0.055	1.277	8.768	0.067

Items are located in order of difficulty (from high AR to low AR). ^†^ degrees of freedom (620,4); ^*^Bonferroni adjustment with a probability base of 0.01 (p = 0.005 for 20 items); note item 12 of borderline misfit. Included in the model because graphical fit was good and fit conceptual model.

#### Threshold Response options

**Figure 2:**
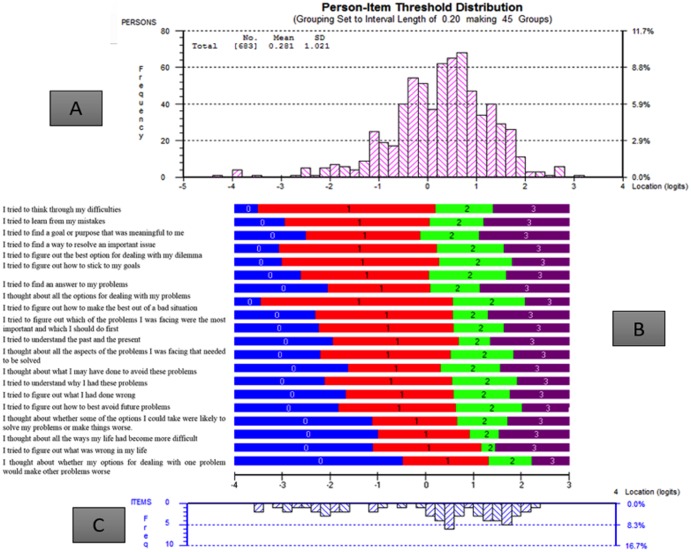
Summary of targeting to the sample of 20 items included in the Analytical Rumination Questionnaire. A. Distribution of items across the measurement continuum in the prototype analytical rumination questionnaire (ARQ). B. Item map showing expected score to each item, with items shown in order of difficulty. C. The location of the 20 items, relative to each other, on an interval scale.

The item response options for 13/22 (59%) items were disordered. As shown in Figure 2b, we rescored disordered items by collapsing the middle category “half of the time” with the second category “some of the time”. After rescoring, statistical and graphical evidence of misfit remained for only two items: “*I thought about all the bad things that could happen to me because of the situation I am in*” (item 13: fit residual  = 5.95; χ^2^ = 48.09, df  = 9, p<0.01); and “*I thought about how others were likely to respond to some of the actions I could take*” (item 9: fit residual  = 4.33; χ^2^ = 29.42, df  = 9, p<0.01). Both items had ICCs well below the theoretical curve, providing evidence of poor discrimination ability. After consultation with two content experts and two clinicians, and revision of conceptual model, the two items were removed.

#### Fit and targeting

Figure 2a shows the distribution of participants along the measurement continuum, ranging from −4.31.to +3.09, reflecting a broad, even spread. As shown in Table 3, overall person fit (i.e., mean person fit residual) was near the targeted level of 0 (mean location  = 0.273, SD  = 1.05) indicating the sample was representative of an expected population distribution. Person locations ranged from −4.50 to +3.20, with only 3 individuals lying outside of the individual fit residual range of −2.5 to +2.5. Item locations and their standard errors are reported in Table 2. Fit of the items was good. Figure 2c illustrates the item threshold range from −3.6 to +2.3 logits which covered 74% of the measurement continuum. Overall item fit was good with a mean (SD) of 0.28 (1.53) as shown in Table 2. Item residuals, χ^2^ fit statistic, and the F-test after Bonferroni correction also were consistent with a reasonable fit.

**Table 3:** Indices of fit to a Rasch model.

ITEM-TRAIT INTERACTION	
Total Item χ	95.26
Total degrees of Freedom	76
Total χ^2^ Probability	0.07
**ITEM-PERSON INTERACTION**	
**ITEM**	
Difficulty	0.00±0.43
Fit Residual	0.28±1.30
**PERSON**	
Measure	0.27 ±1.05
Fit Residual	−0.49 ± 1.91

Figures 2a and 2c show the targeting of the sample to the 20 remaining items, offering evidence of the strong targeting of our sample for evaluating ARQ performance. Scores spanned the range of the scale and were not notably skewed with little evidence of ceiling and floor effects. Of note was that a gap of items was observed >2.3, suggesting that individuals above this range are not as precisely measured as the remainder of the sample (n = 9, <2% of the sample).

#### Person Separation Index

Scale reliability was high (PSI  = 0.87), indicating the items adequately separated this sample along measurement continuum.

#### Unidimensionality

Examination of the eigenvalues from the principal component analysis suggested the presence of two or more subscales. This was also supported by the loadings in the first principle component that showed clear patterns of residuals on successive components, with 5 items with large positive correlations, and 5 others with negative loadings. The first set of items queried the first domain of our conceptual model (understanding the problem), whereas the second set queried the third domain (generating possible solutions to the problem). Evidence from grouping these items together in subtests provided some evidence of multi-dimensionality of borderline relevance, with 8% of the subtests (n = 55) showing significant differences in the estimated differences generated (t = 3.21, p = 0.04).This was a mild deviation from the 5% expected value, warranting further consideration and caution in future testing.

#### Differential Item Functioning

Both graphical and statistical evidence showed the difficulty level of the items was uniform across age, sex, ethnic background, self-report medication use, and time.

## Discussion

The objective of this study was to provide evidence for the conceptual and measurement properties of a new concept of interest in health called *analytical rumination*. Our preliminary results support a set of 20 items, collectively called the Analytical Rumination Questionnaire (ARQ), that cover the full range of our conceptual model of analytical rumination[35]. By application of traditional psychometric and Rasch measurement testing, we have demonstrated that the ARQ is reliable, unidimensional, and meets the criteria for objective rigorous measurement as outlined by the Rasch model. The Rasch model specifically confirmed the presence of a higher-order scale that consisted of 20 items reflecting each of the four theoretical domains previously mapped to the analytical rumination construct (see Figure 1) [35]. From a clinical perspective, our findings support a set of items that suggest a meaningful story of what it may mean to move from “low” analytical rumination to “high” analytical rumination (a fundamental prerequisite of measurement) [60], [63]. For example, Table 1 shows that items on the lower end ask about problem identification (“I tried to think about my difficulties”). As difficulty increases, items capture domains hypothesized to reflect higher analytical rumination such as: identifying and understanding problems, generating possible solutions, evaluating the possible solutions, and learning how to prevent problem recurrence.

Given that recent meta-analyses indicate similar treatment efficacy for cognitive therapies (i.e., psychotherapy) and antidepressant medications [23], [28], the development of the ARQ is timely. The ordering of the items supports the construct validity of analytical rumination, and could possibly be used as a guide to understand how individuals progress from problem identification to the problem resolution. At the level of primary care, family physicians or other health professionals may be able to use this information to engage in a dialogue with patients who experience depressive symptoms. We suggest that the effective assessment and treatment of depression could include helping patients (1) identify of a problems/stressor, (2) prioritize aspects that need solving, (3) identify potential solutions and plans for implementation, and (4) develop a plan to prevent further recurrence of the triggering problem. We hope that clinicians will use this perspective of the potentially adaptive aspects of depression in order to inform treatment. The evolutionary perspective suggests that there are many different aetiological pathways to the diagnostic symptoms of depression, each of which may be best suited to a different treatment strategy. One pathway is the functioning of adaptations designed to promote analytical rumination. The ARQ may be used to identify such cases, and to design personalized interventions that help patients make progress toward the resolution of their triggering problem.

The ARQ currently offers a quick and easy way to assess the stage and progress of a patient's problem-solving analysis. Interventions may therefore be tailored and personalized according to a patient's practical needs to resolve precipitating problems, rather than solely to treat symptoms. For example, psychiatrists and psychologists have developed several treatment strategies that may be effective at the low end of the ARQ spectrum, where patients have not yet identified aspects of their problems or the best solution. Current evidence-based interventions such as “exposure-based” therapies, mindfulness, and problem-solving therapy [70]–[75], that work to increase awareness of problems and reduce avoidance of stressors, may provide an alternative option for care for a population that is difficult to treat with medication alone. At the higher end of the spectrum, there may be more of a role for allied health professionals (e.g., occupational therapy) with expertise in goal-orientated cognitive therapies to help individuals who have identified the problem and goal, but are having difficulty implementing their plan of action. The ARQ developed in the present study will be an invaluable tool for future research to understand the effectiveness of these types of interventions.

From the original 22 candidate items, we found that 2 of the items functioned poorly. Further investigation of the anomalies revealed that each item consisted of more than one question per item (items 9 and 13). We removed these two items since the item locations were close to other items on the scale, and were conceptually redundant. Rasch analysis also revealed inherent problems in the initial 5 option response scale. Upon reflection, we propose two possible explanations for the aberrant behaviour of this scoring structure. First, there may have been too many response options for the target population. A second possibility is that the scoring options were confusing because the categories included both qualitative (all the time, some of the time, etc.) and quantitative (half of the time) response options. Examination of the category frequencies and provided evidence that an optimal scoring structure for this scale would favour four response categories. The anomaly revealed in the scoring structure was resolved upon collapsing the middle category “half of the time”, with the preceding category “some of the time.”

### Limitations of our study

The intended context of use for this scale is adults who experience depressive symptoms. For exploratory purposes, we began our conceptual and measurement testing with young adults in a university setting. Our preliminary results show that the ARQ targets this population very well and has excellent person separation. This sample may not generalise to patients who meet formal diagnostic criteria for clinical syndromes. However, as it currently stands, the ARQ may be useful in a primary care setting, where subclinical symptoms are often encountered. Future work in understanding this concept further in people with depression should include an iterative process of both qualitative and quantitative work to ensure the ARQ items are fit for purpose and measure what they purport to measure.

## Conclusion

Our study provides preliminary results for a set of 20 items (Analytical Rumination Questionnaire) that collectively can be used as a reliable and valid instrument for the quantification of analytical rumination. The ARQ provides a starting point to provide insight into the conceptual underpinnings and measurement of analytical rumination for potential application in the self-management and clinical treatment of depression. Future analyses will further assess the construct validity of the scale by assessing the performance of the items in a clinical population of people with depression.

## Supporting Information

Appendix S1 **Candidate items for the Analytical Rumination Questionnaire.**
(DOCX)
