# Supplementary material for: Measuring the Bright Side of Being Blue: A New Tool for Assessing Analytical Rumination in Depression
Source: PLoS One. 2014 Nov 14;9(11):e112077. doi: 10.1371/journal.pone.0112077 (PMC4232398; doi:10.1371/journal.pone.0112077)
Supplement: Appendix S1 — Candidate items for the Analytical Rumination Questionnaire. (DOCX) [file pone.0112077.s001.docx]

**Appendix A:** Candidate items for the Analytical Rumination Questionnaire

| Item | Item label |
| --- | --- |
| 1 | I tried to understand why I had these problems |
| 2 | I tried to figure out what I had done wrong |
| 3 | I thought about what I may have done to avoid these problems |
| 4 | I thought about all the ways my life had become more difficult |
| 5 | I thought about all the aspects of the problems I was facing that needed to be solved |
| 6 | I thought about all the options for dealing with my problems |
| 7 | I tried to figure out the best option for dealing with my dilemma |
| 8 | I tried to figure out which of the problems I was facing were the most important and which I should do first |
| 9 | I thought about how others were likely to respond to some of the actions I could take. |
| 10 | I thought about whether some of the options I could take were likely to solve my problems or make things worse. |
| 11 | I thought about whether my options for dealing with one problem would make other problems worse |
| 12 | I tried to figure out how to make the best out of a bad situation |
| 13 | I thought about all the bad things that could happen to me because of the situation I am in. |
| 14 | I tried to figure out how to best avoid future problems |
| 15 | I tried to figure out what was wrong in my life |
| 16 | I tried to learn from my mistakes |
| 17 | I tried to figure out how to stick to my goals |
| 18 | I tried to find an answer to my problems |
| 19 | I tried to find a goal or purpose that was meaningful to me |
| 20 | I tried to find a way to resolve an important issue |
| 21 | I tried to understand the past and the present |
| 22 | I tried to think through my difficulties |
